# Lipid Droplets and Their Autophagic Turnover via the Raft-Like Vacuolar Microdomains

**DOI:** 10.3390/ijms22158144

**Published:** 2021-07-29

**Authors:** Muhammad Arifur Rahman, Ravinder Kumar, Enrique Sanchez, Taras Y. Nazarko

**Affiliations:** 1 Department of Biology, Georgia State University, Atlanta, GA 30303, USA; mrahman27@gsu.edu (M.A.R.); esanchez39@gatech.edu (E.S.); 2 Department of Obstetrics, Gynecology and Reproductive Science, University of California, San Francisco, CA 94143, USA; fnu.ravinderkumar@ucsf.edu; 3 School of Biological Sciences, Georgia Institute of Technology, Atlanta, GA 30332, USA

**Keywords:** autophagy, lipid droplets, lipid rafts, lipophagy, microautophagy, microlipophagy, organelle homeostasis, vacuolar microdomains, vacuole, yeast

## Abstract

Although once perceived as inert structures that merely serve for lipid storage, lipid droplets (LDs) have proven to be the dynamic organelles that hold many cellular functions. The LDs’ basic structure of a hydrophobic core consisting of neutral lipids and enclosed in a phospholipid monolayer allows for quick lipid accessibility for intracellular energy and membrane production. Whereas formed at the peripheral and perinuclear endoplasmic reticulum, LDs are degraded either in the cytosol by lipolysis or in the vacuoles/lysosomes by autophagy. Autophagy is a regulated breakdown of dysfunctional, damaged, or surplus cellular components. The selective autophagy of LDs is called lipophagy. Here, we review LDs and their degradation by lipophagy in yeast, which proceeds via the micrometer-scale raft-like lipid domains in the vacuolar membrane. These vacuolar microdomains form during nutrient deprivation and facilitate internalization of LDs via the vacuolar membrane invagination and scission. The resultant intra-vacuolar autophagic bodies with LDs inside are broken down by vacuolar lipases and proteases. This type of lipophagy is called microlipophagy as it resembles microautophagy, the type of autophagy when substrates are sequestered right at the surface of a lytic compartment. Yeast microlipophagy via the raft-like vacuolar microdomains is a great model system to study the role of lipid domains in microautophagic pathways.

## 1. Introduction

Autophagy is defined as the regulated degradation of dysfunctional, damaged, or surplus cellular components and invading foreign entities within vacuoles or lysosomes to restore cellular homeostasis [1]. The phenomenon of autophagy and its associated pathways and key players are conserved from unicellular yeast to multicellular vertebrates, including mammals, such as humans [2]. The conservation of key autophagic proteins across the eukaryotic domain suggests that eukaryotic cells might have acquired this trait quite early during evolution [3]. Over the years, studies using different model systems have shown that autophagy is more than just a degradative or catabolic process. In light of recent studies, it is well established that autophagy is involved in diverse cellular processes such as growth, development, morphogenesis [4], programmed cell death [5], and sexual reproduction [6,7,8]. Autophagy is also frequently associated with several pathologies, including cancer [9], diabetes [10], microbial infections [11], neurodegenerative diseases [12], and so on. The list of autophagic cargos is growing rapidly with the identification of new cellular components that are degraded in the vacuoles/lysosomes, and the associated autophagic pathways are named accordingly [13]. Discussion of the fine details of autophagy and numerous selective autophagic pathways along with the proteins, lipids and membranes involved is beyond the scope of this review and can be found elsewhere [14]. The focus of the present review is on lipid droplets (LDs) and their autophagic degradation in the vacuole, known as lipophagy. We will summarize the information gleaned from studies using yeast along with some highlights from other systems. We will also discuss the different facets of LD homeostasis, including LD biogenesis, fusion/fission, contact sites, and degradation. At the end, we will provide our conclusions and future perspectives, including open questions for further studies of lipophagy in yeast.

## 2. Lipid Droplets

LDs, also referred to as lipid bodies, oil bodies, or adiposomes, are intracellular cytosolic organelles that are present in almost all eukaryotic cells studied to date. This includes cells of algae, fungi, plants, and animals, as well as prokaryotic cells [15,16,17]. LDs are cellular structures that store neutral lipids, primarily triacylglycerols (TAGs), and sterol esters (SEs), which make up their hydrophobic core that is enclosed by a phospholipid monolayer [18]. Neutral lipids are stored within LDs for later intracellular use in producing energy and membranes during cellular starvation [19,20]. LDs were once thought as inert structures for lipid storage. However, the identification of proteins on and within LDs and involvement of LD-associated proteins in its metabolism allows to view LDs as dynamic cellular organelles. Now, LDs are considered more than just fat deposits. They were shown to be involved in diverse cellular processes, including protein, protein complex and RNA storage [21,22], protein degradation, and microbial pathogenesis [23]. The presence of ribosomes on LDs points towards the possibility of protein translation at them [22]. LDs dynamically interact with other organelles to complete numerous physiological functions [24]. Perturbation in LD metabolism is associated with several pathological conditions, such as obesity, diabetes, lipodystrophy, dyslipidemia, fatty liver disease, cholesterol ester storage disease, atherosclerosis, cancer, and inflammation [25,26]. All this suggests that LDs may have a far bigger role in cell physiology than previously thought. Future research on LD metabolism and homeostasis will help to better appreciate the important roles of LDs not only in normal cell physiology but also in disease conditions.

### 2.1. Structure of Lipid Droplets

Similar to other eukaryotic cells, yeast store fatty acids (FAs) and sterols in the form of TAGs and SEs [27]. Yeast LDs are small spherical organelles consisting mainly of those neutral lipids (TAGs and SEs) along with a small amount of phospholipids and proteins [28]. The highly hydrophobic core formed by TAGs and SEs is surrounded by a phospholipid monolayer containing a well-defined set of proteins [29]. These two most prominent classes of neutral lipids are synthesized by the two TAG synthases (Dga1 and Lro1) and two SE synthases (Are1 and Are2) [30]. LDs isolated from yeast are rather homogeneous in size, ranging from 300 nm to 1 μm in diameter, although the size of the LDs in cells of other species may range from 1 to 100 μm (for example, in case of white adipocytes). Under normal conditions, the size of the LDs in non-adipocyte cells rarely goes beyond 10 μm [31]. The size, number, and distribution of LDs are highly dependent on the physiological state of a cell [32]. For example, the budding yeast, *Saccharomyces cerevisiae*, under normal growth conditions (logarithmic phase) possesses fewer and smaller LDs, while the cells under nutrient starvation conditions (late logarithmic or early stationary and stationary phase) have more numerous and bigger LDs [33]. In the late logarithmic/early stationary phase of growth, most LDs fall into a rather narrow 350–450 nm size range, largely independent of their lipid composition [30]. Composition of media, especially the percentage of sugar, and presence of lipids (e.g., oleate) also affect the number, size, and distribution of LDs in yeast [34]. The surface of LDs is decorated by proteins that are involved in the regulation of lipid metabolism. In *S. cerevisiae*, around 45 proteins are known to be present on the surface of LDs. Most of these proteins play important roles in lipid metabolism, LD homeostasis, and other cellular functions, such as storage and degradation of protein aggregates [15,22]. Similar to other cellular organelles, the proteomic analysis of LDs suggests that LD proteins may change significantly both in terms of their identity and quantity [35]. Table 1 shows the bona fide LD-associated proteins in *S. cerevisiae*, most of which were validated in [36]. It is important to mention that composition of LDs in terms of lipids and proteins vary significantly between the LDs of yeast and mammals, and as one moves from yeast to mammalian cells, the number of LD-associated proteins increases [37]. However, this is expected due to the more complex nature of mammalian cells.

### 2.2. Biogenesis of Lipid Droplets

**Figure 1:**
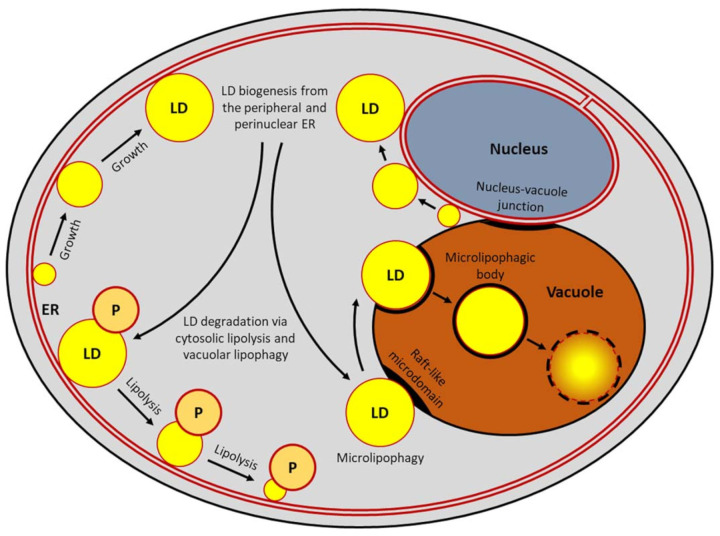
Pathways that contribute to lipid droplet homeostasis in yeast. Lipid droplet (LD) homeostasis is the sum of the anabolic (LD biogenesis from the peripheral and perinuclear endoplasmic reticulum (ER)) and catabolic (LD degradation via cytosolic lipolysis and vacuolar lipophagy) processes. Under nutrient-rich conditions and absence of stressors, yeast cells possess a few small LDs associated with the ER. Starvation for carbon or nitrogen source induces LD biogenesis from the ER membranes, especially the nucleus–vacuole junction. The LDs grow and remain associated with the ER. During prolonged starvation, they can either be degraded in the cytosol by the LD-associated lipases or undergo microlipophagy and degradation in the vacuole by the vacuolar lipases and proteases. During lipolysis, LDs can be associated with peroxisomes where fatty acids from LDs are metabolized via β-oxidation. Microlipophagy involves vacuolar docking of LDs at the newly formed raft-like microdomains followed by internalization of LDs via invagination and scission of the vacuolar membrane, and formation of the intravacuolar microlipophagic bodies that are subsequently disintegrated by the vacuolar hydrolases. P, peroxisome.

Where do LDs come from? Are they autonomously replicating organelles, such as mitochondria, or are they the derivatives from another organelle, such as peroxisomes? The current model of LD biogenesis suggests that LDs are derived from the endoplasmic reticulum (ER) [38,39,40] (Figure 1). The ER is a site where LDs are formed. After maturation, they might still remain connected to the ER or bud off and separate from the ER membrane entirely [41,42]. In recent years, a lot of models were proposed how LDs are derived from the ER or a similar bilayer. In budding yeast, the nucleus–vacuole junction (NVJ) is an important site of LD production (Figure 1). This spatially coordinated ER–vacuole contact site physically expands in response to metabolic stress and serves as an LD factory [43]. It is observed that LD clustering is regulated by the NVJ-associated protein, Mdm1 [44], and LD biogenesis proteins, such as Pah1 [45]. Pah1 is the Mg^2+^-dependent phosphatidate phosphatase, whose activity depends on the Nem1-Spo7 protein phosphatase. Both Pah1 and Nem1 are required for the formation of LDs via coordination of neutral lipid synthesis [45,46,47]. Pah1 provides the precursor diacylglycerol as a substrate for two diacylglycerol-acyltransferases, Dga1 and Lro1 [48], which catalyze the final step in TAG synthesis. In the absence of Dga1 and Lro1, the two sterol acyltransferases, Are1 and Are2, can generate only a minor fraction of TAGs [29,49,50,51]. The yeast homologue of human BSCL2 (also known as seipin), Sei1 (also known as Fld1), physically interacts with the ER membrane protein, Ldb16, and forms a complex involved in LD biogenesis. Yeast counterparts of the fat storage inducing transmembrane protein 2 (FITM2; also known as FIT2), Scs3 and Yft2, and perilipin, Pln1 (also known as Pet10), are also involved in the biogenesis of LDs from the ER [52,53,54,55].

### 2.3. Induction of Lipid Droplet Biogenesis

Over the last couple of decades, our understanding of LD structure, biogenesis, and degradation through lipolysis and lipophagy have improved dramatically. However, the factors and environmental or cellular cues that trigger LD biogenesis are still poorly defined. It is well known in yeast that starvation or scarcity of essential nutrients, such as sources of nitrogen or carbon, induces LD biogenesis. Since both of these classes of nutrients are directly associated with energy metabolism, imbalance in cellular energy homeostasis might force the cells to proceed to LD biogenesis.

Under plenty of nutrients and in the absence of stress, yeast cells either completely lack or possess very few and small LDs. As sources of nitrogen or carbon become limiting, there is a sudden burst in the biogenesis of LDs. As mentioned above, the presence of lipids, such as oleate, also induces biogenesis of LDs in budding yeast [34]. This observation suggests that not only starvation but perturbations in cellular metabolism or homeostasis can also induce LD biogenesis. It is well supported by recent studies where LD biogenesis was observed when cells are put under different stresses, including nutrient overload, hypoxia, exposure of cells to pro-oxidants, drugs, inducers of ER stress, and ceramides [16,56,57,58,59,60,61,62,63]. Cellular stress that induces autophagy also induces the biogenesis of LDs [64,65,66]. Mitochondrial dysfunction and stress associated with cell death are also known to induce LD biogenesis [67,68,69]. Apart from these, the oxidative stress and stress associated with the ER induce LD biogenesis as well [56,57,61,70,71,72]. Surprisingly, in higher eukaryotes, even inflammation is implicated in the induction of LD biogenesis [26,73]. All these suggest the pleiotropic nature of LDs and how LDs are important in maintaining cellular homeostasis during stress. This again highlights the fact that LD is more than just a lipid and energy hub of cells and may have a far bigger role in cellular physiology, which needs to be investigated further. Since different cellular stresses induce LD biogenesis, there must be a common point(s) where all these stress-related pathways converge and connect with the pathways involved in LD biogenesis. This will be both a challenge and an opportunity for the people working in the fields of stress biology and LD homeostasis.

### 2.4. Fusion and Fission of Lipid Droplets

As mentioned above, the number, size, and distribution of LDs vary widely from cell to cell, species to species, and depends on the immediate environment surrounding the cells and their physiological state. However, the number and size of LDs are also affected by the process of LD fusion and fission. Therefore, it is important to have a look at these two aspects of LD biology.

Currently, it is believed that LDs can share their neutral lipids by two different mechanisms. One such mechanism is referred to as Ostwald ripening. In the Ostwald ripening model, small LDs disappear in favor of bigger LDs, thereby minimizing the surface area to volume ratio of the hydrophobic lipids. In this mechanism, lipids are transferred from smaller to larger LDs by simple diffusion [40]. This type of LDs exchange was observed during differentiation of adipocytes [74]. The close apposition of LDs for lipid transfer is mediated by the CIDE family of proteins, including CIDEC (also known as Fsp27), which is involved in the fusion of LDs [75,76,77]. In another mechanism, two LDs come together and merge via coalescence [40]. It is believed that the phospholipid monolayer prevents LD fusion. However, under certain physiological conditions, the composition of the phospholipid monolayer changes and paves the way for the merging of LDs [78,79]. The pharmacological agents that can force the LDs to coalesce are also available [80]. Over the years our understanding of LD fusion improved significantly, and newer proteins involved in LD fusion are regularly being identified in different model systems.

At present, our knowledge of LD fission is very limited and only a few studies are available. A first observation of LD fission was made in mammalian cells in 3T3-L1 adipocytes [81]. Later, Long and co-workers showed the fission of LDs in the fission yeast, *Schizosaccharomyces pombe*, using video and electron microscopy [82]. However, other studies proposed that micro-LDs (which the authors of previous studies might have called the products of LD fission) are the result of cytosolic lipolysis that degrades larger LDs into smaller LDs [83,84]. A recent study in adipocytes showed that formation of small or micro-LDs requires TAG synthase, DGAT1, and protects the ER from lipotoxic stress [85].

### 2.5. Contact Sites of Lipid Droplets

The ER, the birthplace of LDs, maintains a unique relationship with its daughter organelles throughout their lifetime (Figure 1). The enzymes involved in the biosynthesis of TAGs and SEs are localized in the ER, supporting the hypothesis that LDs emerge from the ER. Biogenesis of a large fraction of the LD surface proteins requires their initial insertion into the ER membrane and only later they can transfer to the LDs. In *S. cerevisiae* cells, mature LDs remain in contact with the ER [41,86]. The LDs form contact sites with all regions of the ER (peripheral and perinuclear) during logarithmic growth when nutrients are present. The integral membrane protein, Sei1, localizes to the ER–LD contact sites and its complex with Ldb16 is required for the ER–LD interaction [55,87].

In addition, LDs form contact sites with the vacuole in *S. cerevisiae* (Figure 1). The vacuolar RAB7A-like protein, Ypt7, partially co-localizes with LDs [88]. Cells lacking Ypt7 show the increased number of LDs and abnormal LD morphology. These observations are consistent with a block of the nitrogen starvation-induced lipophagy reported for both *S. cerevisiae* and *Komagataella phaffii* (former *Pichia pastoris*) cells without Ypt7 [34,89]. The contacts of LDs with the vacuole depend on the nutritional state of the cell. During the diauxic shift when cells run out of glucose, LDs start to concentrate adjacent to the NVJ [33,90]. In the stationary phase, LDs move away from the NVJ and start to encircle the vacuole. When cells are facing deep starvation, LDs enter the vacuole for recycling (see also Section 3 below) (Figure 1).

Apart from the physical interactions of LDs with the ER and vacuole, several studies showed a close association of LDs with peroxisomes [91,92,93]. The associations between the LDs and peroxisomes occur via the extension of the peroxisomal membrane structures, called pexopodia [92]. These membrane extensions are densely populated by the enzymes involved in the β-oxidation of fatty acids (peroxisomes are the sole site of β-oxidation in yeast). The collaboration between the LDs and peroxisomes is common during lipid degradation and nutrient scarcity [94]. Interestingly, the M1 isoform of spastin (SPAST) on the LD surface interacts with the ATP-binding cassette subfamily D member 1 (ABCD1) on the peroxisomal membrane and recruits the endosomal sorting complex required for transport III (ESCRT-III) components for fatty acid transfer from the LD to the peroxisome in mammalian cells [95]. Another study found that the physical interaction between the LDs, peroxisomes, and peroxisomal biogenesis factor 5 (PEX5) are important for recruitment of the patatin-like phospholipase domain containing 2 (PNPLA2; also known as ATGL) to the LDs and their cytosolic degradation by lipolysis [96], suggesting that the LD–peroxisome association is common in eukaryotic cells (Figure 1). In addition, LDs and peroxisomes have overlapping organelle biogenesis steps (reviewed in [97]). For example, the LDs and Pex14-containing pre-peroxisomal vesicles in yeast are formed from the same region in the ER shaped by Pex30 and Pex31 proteins [98,99].

It was also observed that the LDs interact with the mitochondria by the bimolecular fluorescence complementation assay in *S. cerevisiae* [100]. The LD proteins, Erg6 and Pln1, are the most prominent players here that interact with many mitochondrial and peroxisomal proteins. In addition, the LD-associated protein, Ldo16 (also known as Osw5), might interact with a few mitochondrial proteins as well. While the interactions of LDs with the ER, vacuole, and peroxisomes are understandable, why LDs bind mitochondria in yeast remains an open question.

## 3. Lipid Droplet Turnover

Like other cell components, LDs follow the cycle of biogenesis and turnover. Before 2009, cytosolic degradation of LDs by lipases (lipolysis) was the only known pathway for cellular degradation of LDs [101]. In 2009, the second pathway of LD breakdown in the lysosomes via autophagy was identified in rat hepatocytes [66]. So far, lipolysis and selective autophagy of LDs or lipophagy remain the only known pathways for cellular degradation of LDs (Figure 1). Enzymes and signals involved in lipolysis have been worked out in detail in both yeast and mammalian cells. The detailed discussion of lipolysis is outside the scope of this review and can be found elsewhere. Here, we will discuss lipophagy with a special focus on microlipophagy via the raft-like vacuolar microdomains as a dominant form of lipophagy in yeast. However, it is important to mention that a prevalent mode of lipophagy in mammalian cells is macrolipophagy, and that mammalian LD degradation by both lipolysis and macrolipophagy also involves the chaperone-mediated autophagy [102,103]. Our understanding of the crosstalk between lipolysis and autophagy is rather limited in yeast and only a few studies are currently available [104,105,106]. Interestingly, the lack of vacuolar lipase, the AuTophaGy-related 15 (Atg15), leads to increased mobilization of LDs via lipolysis [106]. A better understanding of this crosstalk is necessary for a full picture of LD catabolism.

### 3.1. Microlipophagy

In yeast, two main types of autophagy exist, macroautophagy and microautophagy, which can act either selectively or non-selectively. Both degrade the cytoplasmic components, including organelles, that became dysfunctional, damaged, or in surplus. During macroautophagy [107,108], the newly generated autophagic membrane encapsulates the cellular constituents into the double-membrane vesicle, autophagosome. The origin of the autophagic membrane is controversial and several sources have been proposed. It may be derived from the ER, mitochondria, or plasma membrane [109,110]. Finally, autophagosomes fuse with the vacuole and their cargoes are digested by vacuolar hydrolases. In contrast to macroautophagy, microautophagy degrades cargoes via the direct vacuolar engulfment, with or without the formation of an additional autophagic membrane [111,112,113]. Microautophagy can selectively degrade various specific cargos, such as the nucleus, peroxisomes, mitochondria, ER, and LDs, in the yeasts *S. cerevisiae* and *K. phaffii* [34,114,115,116,117]. The selective microautophagy of LDs or microlipophagy is an autophagic process that delivers specifically the LDs from the cytosol to the vacuole for degradation and recycling and is the main mode of lipophagy in yeast (Figure 1).

### 3.2. Induction of Microlipophagy

Contrary to mammalian lipophagy, which can be induced by rapamycin [66], treatment with rapamycin fails to induce lipophagy in yeast [118]. However, it can be induced by numerous other ways, such as acute nitrogen starvation [34,89], carbon starvation (both acute [118,119] and gradual [33,89,120]), and acute nitrogen–carbon starvation combined [121]. To achieve acute nitrogen starvation, *S. cerevisiae* and *K. phaffii* cells are transferred to the medium without a source of nitrogen [34,89]. For acute carbon starvation, cells of *S. cerevisiae* are switched from a medium with 2% glucose to a medium with 0.4% or 0.5% glucose [118,119]. Gradual carbon starvation of *S. cerevisiae* cells is achieved in the medium with 2% glucose after a diauxic shift when the cells fermented all the glucose in the medium to ethanol and started using ethanol as a less preferable carbon source. Gradual carbon starvation continues into the stationary phase when the cells of the budding yeast run out of ethanol. In this case, it is called stationary phase lipophagy [33,120]. Stationary phase lipophagy can also be induced in *K. phaffii* without the fermentation of glucose to ethanol and diauxic shift [89]. Finally, acute nitrogen–carbon starvation is created by transferring the cells to the medium without both nitrogen and carbon sources [121].

The ER stress (due to phospholipid imbalance or treatment of cells with dithiothreitol or tunicamycin) also leads to microlipophagy in yeast [122,123]. To induce lipophagy by phospholipid imbalance, researchers use the *cho2* mutant deficient in the phosphatidylethanolamine methyltransferase as it is unable to synthesize the phosphatidylcholine and develops the ER stress [122]. A similar mode of microlipophagy, which involves vacuole fragmentation and homotypic fusion of vacuolar compartments, can be induced by dithiothreitol or tunicamycin that, similar to the genetic block of phosphatidylcholine biosynthesis, also induce the ER stress [123]. Overall, growing cells into the stationary phase or transferring them to the medium without nitrogen are the two most common ways to induce lipophagy in yeast. Both conditions induce LD biogenesis and microlipophagy.

### 3.3. Molecular Mechanism of Microlipophagy

#### 3.3.1. Formation of Vacuolar Microdomains as a Prerequisite for Microlipophagy

Before discussing vacuolar membrane reorganization during microlipophagy, it is important to brief the readers on lipid domains. The composition of the cell and organelle membranes (in terms of lipids, proteins, and fluidity) is not uniform. The concept of lipid rafts (in reference to the nanometer-scale membrane regions) or lipid domains (in general) has gained a significant interest and importance. Lipid rafts are the regions in the cell membrane that are rich in cholesterol, sphingolipids, and proteins compared to the surrounding plasma membrane. It is believed that these organized domains of lipids and proteins are important in cell physiology and are involved in diverse cellular functions. However, it is important to mention that lipid rafts were never separated or purified from cell membranes, reflecting their transient nature. Therefore, their existence remains controversial. Still, lipid domains with increased levels of sterols, sphingolipids, and proteins have been detected in the plasma membrane of diverse cells as well as in the membranes of cellular organelles, including lysosomes, Golgi apparatus, etc. Lipid rafts are more ordered and rigid compared to the surrounding region, which is less ordered and more flexible. Lipid rafts are believed to concentrate and organize the membrane-associated proteins involved in cell signaling. A detailed discussion of these transient nanodomains is beyond the scope of this review and can be found elsewhere [124,125,126,127].

Similar to the plasma membrane lipid nanodomains, lipid microdomains have been reported in yeast vacuoles [128,129]. Vacuolar microdomains refer to the two stable micrometer-scale lipid domains, namely, the sterol-rich liquid-ordered domain and sterol-poor liquid-disordered domain, that are formed in the vacuolar membrane under various stress conditions, such as the stationary phase, acute carbon starvation, acute starvation in water, translation inhibition with cycloheximide, weak acid (acetate, pH 5.2), and heat (37 °C) stresses [128]. Remarkably, all 15 vacuolar membrane proteins tested in this study segregated between the two microdomains, with the majority of them being localized to the liquid-disordered domain and only two proteins, Gtr2 and Ivy1p, sitting in the liquid-ordered domain that resembles lipid rafts in the plasma membrane. Further studies added Atg6 and Atg14 (next section), Lam6/Ltc1, Gtr1, Iml1, Tco89, and Nce102 (see below) to the list of raft-like vacuolar domain-associated proteins [33,130,131,132].

The initial study has also established the first molecular requirements for the vacuolar microdomain formation: (1) ergosterol or its precursors (e.g., in *erg5* and *erg6* mutants); and (2) the Fab1, Mpk1, Nem1, Sec18, and Vps4 proteins [128]. Interestingly, Fab1, Mpk1, Nem1, and Vps4 were needed for microdomain formation in the stationary phase, during acute starvation in water, and under weak acid stress. However, the cycloheximide-induced domain formation required only Fab1 and Vps4 out of these four proteins, implying a partially different molecular mechanism. The subsequent studies identified other proteins necessary for the raft-like vacuolar domain formation, such as Lam6, the ER membrane protein that localizes to the ER–vacuole contact sites (including NVJs) via its interaction with the vacuolar membrane protein, Vac8 [130,133]. Surprisingly, Lam6 was required for microdomain formation during acute carbon starvation and cycloheximide treatment, but not under weak acid stress, suggesting, once again, that microdomain formation is achieved somewhat differently under different stress conditions. Since Lam6 has a sterol-transfer activity in vitro and localizes to the sterol-rich vacuolar domains in vivo, it might have a direct role in their expansion. This is also supported by the fact that driving Lam6 to the ER–vacuole contact sites (by abolishing its localization to the ER-mitochondria contacts) increases the number of cells with vacuolar microdomains under normal growth conditions [130]. In addition, overexpression of Lam6 leads to expansion of all the membrane contact sites containing it, namely, ER–mitochondria, vacuole–mitochondria contacts, and NVJs, causing micronucleophagy [133]. Therefore, Lam6 can potentially contribute to the expansion of the vacuole–LD contact sites and microlipophagy.

Another raft-like vacuolar domain-associated protein that regulates the formation of this domain is Nce102 [132]. This protein partially re-localizes from the lipid rafts in the plasma membrane to the raft-like microdomains in the vacuolar membrane via endocytosis and the multivesicular body pathway when cells enter the stationary phase. Interestingly, Nce102 negatively regulates the size of the liquid-ordered domains because in the *nce102* mutant, they are substantially larger correlating with the increased intravacuolar degradation of the liquid-disordered domain marker, Vph1 [132]. Regarding other non-Atg raft-like domain-associated proteins, they were not implicated in microdomain formation. For example, Iml1 was not required for microdomain formation during cycloheximide treatment [131], while Gtr1, Gtr2, Ivy1, and Tco89 were dispensable for microdomain formation at least during the acute starvation in water [128]. Therefore, not all the proteins that localize to the raft-like vacuolar domains are involved in their biogenesis.

Interestingly, the raft-like vacuolar microdomains contain the phosphatidylinositol 4-phosphate (PI4P) nanoclusters, and these clusters form only in the cytosolic leaflet of the vacuolar membrane that comes in direct contact with the cytosolic LDs in the stationary phase [121,134]. Consistently, the PI4P appears exclusively in the cytosolic (inner) leaflet of the microautophagic bodies that accumulate in the vacuole lumen in both the stationary phase and during the acute nitrogen–carbon starvation [121]. The PI4P nanoclusters of the raft-like vacuolar microdomains play an important role in microautophagy, because the mutants of the yeast phosphatidylinositol 4-kinases (PI4Ks), Stt4 and Pik1, have much smaller number of microautophagic bodies in both the stationary phase and during the acute nitrogen–carbon starvation [121]. However, it is not clear if the PI4Ks affect the raft-like vacuolar domain formation.

During the stationary phase lipophagy, the internalization of LDs by the vacuole depends on their association with the sterol-rich vacuolar microdomains [33]. Previously, it was shown that rapamycin and acute nitrogen starvation do not induce vacuolar domain formation [33,128], but recent studies suggest that such microdomains are formed during the acute nitrogen starvation as well [135]. In the absence of intact vacuolar microdomains, LDs are unable to contact the vacuole and remain associated with the ER. As mentioned above, sterols are important for vacuolar microdomain formation and maintenance [33], and sterol transport by the Niemann–Pick type C (NPC) proteins, Ncr1 and Npc2, from the lumen to the membrane of the vacuole is essential for microdomain formation and microlipophagy [135]. Npc2 plays an especially important role in the formation of raft-like microdomains both in the stationary phase and during acute nitrogen starvation [135]. Initially formed raft-like vacuolar microdomains facilitate microlipophagy, which provides an additional source of sterols that have been stored in LDs by Are1 and Are2 in the form of SEs. This fuels further microdomain formation [33,135]. Interestingly, the components of the Sei1 complex, Ldb16 and Ldo16/Ldo45, are necessary for efficient lipophagy in the stationary phase and are involved in microdomain formation [136].

#### 3.3.2. The Requirement of Autophagic Proteins for Vacuolar Microdomain Formation and Microlipophagy

Previously identified selective microautophagy pathways require Atg-proteins for their cargo degradation [137,138] and the microautophagy of LDs (microlipophagy) is not an exception, even though it does not involve the formation of an autophagic membrane. Microlipophagy needs the entire core autophagic machinery, including the Atg1 kinase complex (except Atg11), phosphatidylinositol 3-kinase (PI3K) complex I components, Atg9 cycling system, and Atg8/Atg12 conjugation systems under various conditions tested [33,34,118]. In early studies of yeast microautophagy, it was shown that vacuolar invaginations and uptake of solutes during acute nitrogen starvation depend on the core Atg-factors both in vitro and in vivo [112,113]. However, it was proposed that the role of Atg-factors is indirect. They might support membrane influx via the fusion of the Atg-built autophagosomes with the vacuolar membrane, the influx necessary to sustain the formation of vacuolar invaginations. Indeed, a recent study showed that the core Atg-factors, Atg7 and Atg8, are required for vacuolar microdomain formation during acute nitrogen starvation-induced lipophagy [135]. Unexpectedly, the core autophagic machinery was dispensable for the microautophagy after diauxic shift [120]. Its role was also different during the stationary phase lipophagy where it was necessary for proper vacuolar localization of the NPC proteins and microdomain maintenance (not formation) [33,135]. Finally, the study in *K. phaffii* showed that while the acute nitrogen starvation-induced lipophagy strictly depends on the core autophagic machinery, only Atg6 is essential for lipophagy in the stationary phase [89]. The rest of the core Atg-factors were only partially required. Consistently, Atg6 (and, surprisingly, Atg8) was essential for the vacuolar microdomain formation in the stationary phase [33] (note that Atg8 was not required for this during the acute starvation in water [128]).

The requirement of autophagic selectivity factors for lipophagy might be different under different conditions as well. While the acute nitrogen starvation-induced lipophagy requires the vacuolar membrane protein, Vac8 [34], the stationary phase lipophagy needs the phosphatidylinositol 3-phosphate (PI3P) binding protein, Atg21, and mitophagy receptor, Atg32 [33]; the acute carbon starvation-induced lipophagy depends on the cytoplasm-to-vacuole targeting (Cvt) receptor, Atg34 [118], and the phospholipid imbalance-induced lipophagy is regulated by the nucleophagy receptor, Atg39 [122]. However, the above requirements of Vac8 and Atg32 were missing in *K. phaffii* [89], suggesting that they might be species specific. The proteins specific for lipophagy and not involved in other autophagic pathways are still unknown. The selectivity factors implicated in lipophagy so far participate in two or more types of selective autophagy. For example, Atg21 is needed for Cvt, lipophagy, mitophagy, nucleophagy, and pexophagy [33,137,139,140,141]. Therefore, the lack of lipophagy-specific selectivity factors is the key gap in our understanding of yeast microlipophagy. Unfortunately, they cannot be identified by a simple screening of known *atg*-mutants, since those mutants are already deficient in at least one other autophagic pathway, as demonstrated above.

The intravacuolar lipase, Atg15, was at least partially required for the internalization/degradation of LDs during microlipophagy under all conditions [33,119,139,140]. In contrast, there were some exceptions as to the role of other Atg-proteins under specific lipophagy conditions. For example, while the vacuolar membrane protein, Atg22, which is responsible for the efflux of amino acids from the vacuole, was essential for stationary phase lipophagy [33], it was dispensable for the acute carbon starvation-induced lipophagy [118]. However, even more important were the following exceptions: (1) Atg1 was not essential for the microlipophagy after diauxic shift [120]; (2) Atg7 was dispensable for the phospholipid imbalance-induced lipophagy [122]; and (3) Atg1 and Atg8 were not required for the dithiothreitol- and tunicamycin-induced lipophagy [123]. Therefore, mounting evidence suggests that under certain conditions, such as gradual carbon starvation (see also above) and ER stress, the core Atg-machinery is non-essential for lipophagy in yeast.

The PI3K complex I components, Atg6 and Atg14, deserve special attention. They were strongly required for lipophagy under all conditions tested [33,34,89,118]. However, the main subunit of the complex carrying PI3K activity, Vps34, was shown to be essential only for the acute nitrogen starvation-induced lipophagy [34]. Furthermore, while Atg6 and Atg14 localized to the raft-like vacuolar microdomains under gradual and acute carbon starvation, Vps34 did not [33,118], suggesting that Atg6 and Atg14 might be playing a different role during carbon starvation not related to PI3P production (their main function under nitrogen starvation conditions). Moreover, Atg6 localized exclusively at the vacuolar membrane under both gradual and acute carbon starvation independently of Snf1, whereas Atg14 fully re-localized from the ER exit sites to the vacuolar membrane only during acute carbon starvation in a Snf1-dependent fashion. Consistently, Atg6 was strongly required for the vacuolar microdomain formation under both carbon starvation conditions, while Atg14 and Snf1 were essential and partially required, respectively, for microdomain formation only during acute carbon starvation [118]. Since the raft-like vacuolar microdomains are larger during acute carbon starvation, their formation might require additional components, such as Atg14. It is important to note that despite being dispensable for microdomain formation during gradual carbon starvation, Atg14 is still required for their maintenance, similar to other core and selective (Atg21 and Atg32) Atg-factors in the stationary phase [33].

#### 3.3.3. Role of ESCRT Machinery in Microlipophagy

The ESCRT, which consists of membrane-associated proteins involved in membrane scission events, is also involved in microlipophagy. Interestingly, both Atg-dependent and -independent pathways of microlipophagy depend on the ESCRT machinery [123]. It was reported that the microautophagy of the vacuolar membrane protein, Vph1, does not require the core Atg-proteins after the diauxic shift and mainly relies on the ESCRT machinery. Microlipophagy under those conditions is also dependent on the ESCRT component, Vps27, and its interaction with clathrin [120]. To encapsulate the associated LDs, the ESCRT machinery localizes to and drives the invagination of the vacuolar membrane [120,122]. The direct role of ESCRT and vacuolar membrane fusion are also evident in the ER stress-induced microlipophagy [123], where the ESCRT proteins are recruited to the neck of the vacuolar membrane invagination to rearrange the vacuolar membrane, capture the LDs, and create a force to the membrane for its internalization [123]. On the other hand, the ESCRT machinery negatively regulates LD degradation during acute carbon starvation [119]. In the ESCRT mutants, Atg14 plays an important role in the degradation of LDs. It localizes to the vacuolar membrane and might be involved in the formation of vacuolar domains [119].

#### 3.3.4. Role of Nucleus-Vacuole Junction in Microlipophagy

The NVJ is formed by the interaction of the nuclear membrane protein, Nvj1, and vacuolar membrane protein, Vac8 [142]. The NVJ expands under nutritional stress and is linked to lipid homeostasis. LDs are synthesized at the ER in response to various nutritional stresses and NVJ serves as a site of LD budding [43]. Another protein, Mdm1, has a connection with both the ER and vacuole membranes even in *nvj1* mutant cells and plays an important role in the NVJ-associated LD budding [44]. The *NVJ1* mRNA levels increase, and the contact site enlarges in the stationary phase [43]. Since during the stationary phase lipophagy LDs migrate from the perinuclear ER to the vacuolar membrane, NVJ may promote such a movement [90]. Through the concentration and coordination of the molecular machinery at NVJ, this junction may impact the stationary phase lipophagy. However, it was reported that while the deletion of the *NVJ1* gene disrupts the NVJ in yeast [142], it does not compromise the formation of vacuolar microdomains [128].

## 4. Lipid Droplets and Diseases

Cells maintain a fine balance between LD biogenesis and degradation. Defects in either LD biogenesis or degradation have been associated with several pathological conditions. Over the years, research on LD metabolism showed that increased LD accumulation in various types of tissues and organs, such as adipose tissue, liver, and skeletal muscle, is a sign of metabolic syndrome. Perturbations in LD degradation due to defects in either lipolysis or lipophagy leads to important clinical conditions, including neutral lipid storage diseases, obesity, diabetes, and atherosclerosis [143,144]. Enlarged LDs are often observed in the aforementioned tissue and organs under disease conditions. Not only the excessive accumulation of lipids in the cells or body, but also deficiency or complete absence of LDs may lead to a rare lipid-associated clinical condition known as lipodystrophy. The defect at any point in the synthesis of neutral lipids may affect LD biogenesis and result in the degenerative white adipose tissue [145]. This again shows that LDs are not just inert fat deposits and have a bigger role in cell/organism physiology.

## 5. Conclusions and Future Perspectives

Lipophagy degrades LDs in the vacuole/lysosome. Currently, several types of microlipophagy were reported in yeast that are induced by different conditions and utilize different molecular mechanisms to uptake LDs inside the vacuole. The essential roles for a few Atg-proteins and ESCRT machinery were proposed, but future studies are needed to find the exact molecular mechanisms underlying these pathways. Many questions still remain unanswered, for example:

- Is microlipophagy the only morphological mode of lipophagy in yeast?
- Why are microlipophagy mechanisms distinct under different conditions?
- What is the nature of the contact sites between the vacuolar microdomains and LDs?
- What are the exact functions of autophagic proteins in microlipophagy?
- What is the autophagic receptor protein during microlipophagy (if any)?
- What machinery is required to recycle lipids after degradation?
- How extensive is the crosstalk between lipophagy and lipolysis in yeast?
- How does the cell decide whether to proceed for lipolysis or lipophagy?

We hope that future studies will clarify these questions and provide a more complete picture of LD uptake and turnover in the yeast vacuole. However, from the available information and comparison of lipophagy under various conditions, it is already evident that the involvement of autophagic machinery in microlipophagy depends on how it was induced. Whether this is true for other autophagic pathways (e.g., mitophagy, pexophagy, reticulophagy, etc.) remains as yet another open question for future studies and comparisons of these pathways under different conditions. Surely, yeast microlipophagy via the raft-like vacuolar microdomains will continue to provide insights into the roles of lipid domains in the microautophagic pathways of vacuolar degradation and recycling.

## Figures and Tables

**Table 1:** Yeast LD-associated proteins validated in [36] and other studies.

LD-Related Function	Proteins enriched in LDs and/or Co-Localized with LDs
LD biogenesis	Ldb16, Ldo16, Ldo45, Mdm1, Pln1, Sei1
TAG synthesis	Ayr1, Dga1, Pah1, Pgc1, Slc1
Ergosterol metabolism	Erg1, Erg6, Erg7, Erg27, Say1, Srt1
Lipolysis-related	Ice2, Ldh1, Lpl1, Tgl1, Tgl3, Tgl4, Tgl5, Yeh1, Yju3, Ypr147c
FA/phospholipid-related	Eht1, Faa1, Faa4, Fat1, Hfd1, Loa1, Pdr16
Other lipid-related functions	Atf1, Cab5, Nus1, Rer2, Tsc10, Ubx2
LD-related role unknown	Anr2, Yim1, Lds1, Lds2, Rrt8
